# Traditional Chinese medicine to improve rural health in South Africa: A case study for Gauteng

**DOI:** 10.4102/hsag.v27i0.1871

**Published:** 2022-09-27

**Authors:** Zijing Hu, Roy Venketsamy

**Affiliations:** 1 Department of Complementary Medicine, Faculty of Health Sciences, University of Johannesburg, Johannesburg, South Africa; 2 Department of Early Childhood Education, Faculty of Education, University of Pretoria, Pretoria, South Africa

**Keywords:** rural health, alternative medicine, Africans, traditional Chinese medicine

## Abstract

**Background:**

Rural health is a global crisis across different continents. Most of Africa is predominantly rural and is experiencing financial constraints. Medical support and supplies are a dire need in rural Africa. An alternative option to support the rural population is through traditional Chinese medicine (TCM). Studies have highlighted the efficacy and cost-effectiveness of Chinese medicine in improving health.

**Aim:**

This article aims to investigate how rural health can be improved through alternative medicine options, focusing primarily on TCM.

**Setting:**

An identified TCM practice in Gauteng province was selected as the research setting.

**Methods:**

This study adopted a qualitative case study design to explore 10 participants’ views on TCM to improve rural health. The health belief model was used as a theoretical framework, and thematic analysis was used for this study.

**Results:**

Findings revealed that most participants accepted TCM as an alternative medical health care option as it resonated with African herbal medicine. Participants agreed that TCM is cheaper and has no side effects.

**Conclusion:**

There is a lack of appropriate medical service providers in most rural areas, and often, people depend on traditional medicines as a quick remedy. As TCM is cost-effective and has proven to treat numerous ailments successfully, it is recommended that it be further explored as a health care option available to rural populations.

**Contribution:**

This was the first study on South African patients’ views on TCM in the South African context.

## Introduction

Improving rural health is a global phenomenon and has been recognised as a crisis in Africa by many health organisations both internationally and nationally. The availability of affordable and attainable health care services is still a critical challenge in Africa. Before the independence of African states, colonial health care providers supported healthcare in Africa. According to Azevedo (2017), the sovereignty of African states forced colonial health care providers to restrict their support to the people of Africa. Despite traditional African medicine gaining popularity, studies by Mellor (2014) and Thou (2018) revealed that poverty, socio-economic conditions and poor health contributed to the decline of rural health in most parts of Africa. Similar findings were reported by Guo et al. (2017) and Tlou (2018), who asserted that Africa has a significant shortage of health care workers and practitioners. There is a desperate need for the improvement of health services amongst rural communities.

A large part of the African continent is rural, with a high need for medical support and services (Melor, 2014). The shortage of health care workforce, poor health facilities and structures and limited access to diagnostic services affect the improvement of rural health (Guo et al. 2017; Ogunkola et al. 2020). For these reasons, it is crucial to give attention and focus to rural communities to support and improve their health conditions, especially during the coronavirus disease 2019 (COVID-19) pandemic. Both Mellor (2014) and Tlou (2018) believed that to ease the burden of the increasing health crisis in rural Africa, alternative medicine options should be made available to rural communities. This article investigated how rural health can be improved through alternative medicine, focusing primarily on traditional Chinese medicine (TCM). The authors formulated the following research question: *how can rural health be improved through the use of traditional Chinese medicine?* In this article, the challenges of rural health in Africa were addressed. The researchers reviewed several academic papers discussing the benefits and effectiveness of TCM as an alternative form of medicine for rural communities.

Traditional medicine (TM) is defined as (WHO 2013):

[*T*]he knowledge, skill, and practices based on the theories, beliefs, and experiences indigenous to different cultures used in the maintenance of health, prevention, diagnosis, improvement or treatment of physical and mental illness. (pp. 15)

The term TM is used interchangeably with alternative medicine, which refers to foreign health care practices that neither originate from that country’s tradition nor are fully integrated into the health care system (World Health Organization [WHO] 2013). As stated by WHO (2013) and Zhang et al. (2019), TCM is one of the predominant alternative medicine systems that has been widely accepted globally. Traditional Chinese medicine dates back to 475 BC and has proven effective in treating many illnesses in most Asian countries. In this article, the authors adopted a qualitative case study design to investigate how rural health can be improved through alternative medicine. The objectives of this study were to explore participants’ attitudes and perceptions of alternative medicine with a particular focus on TCM.

## Literature review

### Rural health conditions in Africa

Whilst scholars have proposed different definitions of ‘rurality’, there is no consensus on a standardised description accepted universally (Bosworth & Somerville 2014; Gaede & Versteeg 2011). Although each nation has its definition of rural and urban, population size and distance from urban centres are the most critical metrics, according to Bosworth and Somerville (2014). Gaede and Versteeg (2011) further highlighted that some shared characteristics can assist in identifying rural areas, such as the availability of public services (rural health provision) and public amenities. This article defines rural health as the health and health care delivery in rural areas lacking urban characteristics such as amenities and infrastructure.

Rural health in Africa has become a critical matter, and one of the reasons is the lack of health care service providers (Guo et al. 2017; Ogunkola et al. 2020). Ogunkola et al. (2020) agreed with Amzat and Razum (2018) that more than 60% of the population on the African continent lives in rural areas, whilst most professional health care services are located in urban areas. These authors also emphasised that modern health care services and facilities do not exist in many rural regions of Africa. Similar findings have been reported by Harris et al. (2011), who surveyed 4668 participants in South Africa (SA). They agreed that there is a significant shortage of appropriate health care services in most regions. Harris et al. (2011) state that the accessibility of available health care services is still an enormous challenge in rural settings. Against this backdrop, the authors believe that the lack of medical services has significantly affected the health of rural areas. It is therefore necessary to introduce available, uncomplicated and effective medical approaches amongst rural communities.

Amzat and Razum (2018) highlighted that TM is widely applied to primary health in African counties, especially in rural areas. They explained that the reason is that TM is cost-effective, affordable and easily accessible. Traditional medicine is considered an alternative healthcare system in many African countries. Therefore, they argued that it would reduce the burden on healthcare in Africa if implemented widely (Amzat & Razum 2018). It must be borne in mind that TM has many distinct features, including culture, spirituality and experience base (Amzat & Razum 2018). However, it is argued that the involvement of the spiritual aspects in TM practice has limited its implementation in different contexts because it may be perceived as voodooism, ancient healing or pantheism.

### The need for alternative medicine

Amzat and Razum (2018) revealed that, for years, the health burden of primary diseases in rural areas in Africa has contributed to almost 70% of the morbidities and mortalities. They further highlighted that one of the primary challenges is the high cost of health care, especially in rural areas where poverty is more prevalent. Lower costs on medical services are an important reason for the public to choose alternative medicine forms, significantly so when medical costs are escalating along with chronic diseases (Mellor 2014; Tlou 2018; WHO 2013). The researchers support that alternative medicine will reduce the government’s financial burden, especially amongst the rural population during times of dire crisis, for example, the COVID-19 pandemic.

Maroyi (2013) argued that in Zimbabwe, alternative medicine remains the most affordable and acceptable medicine to the public. In Ethiopia, Wassie et al. (2015) revealed that the residents are highly accepting of alternative medicine, which can be confirmed in that 70.9% of participants have used herbal medicine at least once. Similarly, in South Africa, many people in rural areas often consult with traditional or herbal doctors. They have robust belief systems deeply entrenched in their cultural philosophy that traditional healers will heal their illnesses. This can be confirmed by Semenya and Potgieter (2014), who highlighted that TM plays a vital role in the primary health of the rural population in Limpopo, South Africa. Harris et al. (2011) pointed out that one of the challenges in South Africa is the lack of health care service providers, whilst there is an increased need for quality health care services. We note that a similar situation can be seen in other African countries on the continent.

As discussed previously, health care services are not available in many rural areas in African countries because of poor accessibility and affordability. Wardle et al. (2011) argued that alternative medicine plays a vital role in health in rural settings without the availability of conventional health care services. The shortage of conventional health care professionals increases alternative or TM use, as reported by Guo et al. (2017) and Ogunkola et al. (2020). Wardle et al. (2011) asserted that alternative medicine is more likely to be accepted when there is no access to conventional medical assistance. We argue that TCM may be one such solution to improve rural health.

### Importance of traditional Chinese medicine as an alternative medicine

Traditional Chinese medicine is one of the primary forms of alternative medicine worldwide that has a long history of constant clinical practice (Zhang et al. 2019). It is an ancient medical system that treats diseases with different interventions, including Chinese herbal medicine, acupuncture, moxibustion, Chinese therapeutic massage, cupping, food therapy and exercise (Li, Chen & Zhao 2019). Chinese herbal medicine is one of the most common therapeutic modalities in TCM, mainly focusing on using plants to treat various diseases (Zhang et al. 2019). Acupuncture is performed by inserting thin needles on specific points (namely acupoints) in the body to treat various diseases (Li et al. 2019). Moxibustion is another therapeutic technique that burns moxa cones or sticks (*Artemisia argyi*) over acupoints (Cheng 2019). Cupping therapy is achieved by creating negative pressure on the skin, and it is commonly used in TCM practices (Cheng 2019; Li et al. 2019). According to TCM theories, food therapy takes the effects of food on the properties, tastes and therapeutic functions (Li et al. 2019).

Traditional Chinese medicine focuses not only on the treatment of diseases but also on the prevention of diseases. Evidence supporting this view can be found from Kim et al. (2018), who argued that TCM is a recommended treatment for many medical conditions. Traditional Chinese medicine is recommended to be used as an alternative medicine to improve health conditions, according to Lam, Lyu and Bian (2019) and WHO (2013). Traditional Chinese medicine has played a memorable role in the treatment of pandemics in recent years, from severe acute respiratory syndrome (SARS) in 2003 to the COVID-19 outbreak in 2020, as specified by Yang et al. (2020). This opinion is further asserted by Ren, Zhang and Wang (2020), who claimed that remarkable clinical effects have been shown in treatment with TCM. They reported that TCM may increase the clinical cure rate of COVID-19, reduce the percentage of cases that develop into severe conditions and shorten the average length of stay in hospitals.

Many TMs involve the practice of spirit, as revealed by Amzat and Razum (2018). Although the words ‘spirit or spiritual’ are adopted in TCM theories, the original term in TCM is *shen*. This term does not relate to religious discourse or spiritual aspects in TCM, as it refers to the interpretation of the highest level of heaven and earth in theory (Wielander 2017). The researchers of this study agree that this term refers to the mind or psychological perspective. Therefore, according to the WHO, TCM has no spiritual concern, even though TCM is defined and categorised as TM (or alternative medicine). In light of this, there should be no confusion in implementing TCM in rural communities from a spiritual concern (Amzat & Razum 2018).

### Role of traditional Chinese medicine in advancing rural health

Traditional Chinese medicine aims to treat individuals with a holistic approach (Li et al. 2019), including interventions from multiple aspects. These approaches include food therapy (diet), lifestyle, herbal medicine, acupuncture, moxibustion, cupping and Chinese therapeutic massage. Most of these interventions are accessible and affordable, which meet the unique needs of rural health in Africa. Studies have revealed that TCM is practical and cost-effective in treating many different diseases with fewer adverse effects and side effects (Lam et al. 2019; Li et al. 2019). According to a survey conducted by Venzke, Calvert Jr and Gilbertson (2010), in a rural area in the United States, it was reported that acupuncture can successfully assist postmenopausal women, including reducing hot flashes, depression and anxiety. This concurs with Schlabach et al. (2012), who conducted a study in Nepal and revealed that the low cost and effectiveness of TCM use may assist primary health in developing countries, especially in rural areas. Despite the advantages of TCM, Fung and Linn (2015) argued that there is a need for TCM to be explained and proven in an evidence-based approach.

## Theoretical framework

The health belief model (HBM) was adopted in this article as a theoretical framework. This model was developed in the 1950s in the United States and has been widely accepted and applied for health promotion in different populations (Tarkang & Zotor 2015). According to Abraham and Sheeran (2015), the HBM is explained by individuals’ threat perception and behavioural evaluation, which are further interpreted into perceived susceptibility, perceived severity, perceived benefits and perceived barriers. The perceived vulnerability refers to a person’s awareness of diseases, whilst the perceived severity is defined as the individual’s perception of the severity of conditions. The perceived benefits are presented as a person’s knowledge of various actions to prevent or cure diseases. The perceived barriers refer to a person’s understanding of the obstacles to implementing health behaviours. In addition, the cue to action and self-efficacy are included in the model. The action signal refers to both internal and external stimuli, which encourage individuals to accept healthy behaviour. Self-efficacy is defined as the ability to act.

In this article, the researchers focused on rural health challenges and the advantages of implementing TCM as an alternative medicine option for promoting rural health. Health is a holistic process that includes different aspects, such as health, diet and treatments. The HBM guides individuals’ health activities and promotes healthy behaviours.

## Research methodology

Qualitative research is a systematic research technique that studies the meanings, characteristics and understandings of participants (Creswell 2014). The qualitative research approach assists in exploring lived experience of participants (Maree 2020). The study aimed to explore participants’ views, personal experiences and perceptions on how rural health can be improved by using TCM. This approach also allows participants to freely verbalise their experiences and share their experiences of using TCM as an alternative treatment. The interpretive paradigm is a subjectivist epistemology that relies on the researcher’s understanding and comprehension when making sense of participants’ experiences (Yin 2018), allowing the researcher to probe into the responses of the participants to gain a deeper insight into their experiences and perceptions (Creswell 2014).

A single case study design was selected for this study. A case study approach was used as ‘an intensive study about a person, a group of people or a unit, aimed to generalise over several units’ (Gustafsson 2017:2). This method offered an opportunity to explore and make meaning of participants’ experiences, as supported by Creswell (2014) and Yin (2018), who agreed that studying a single case will provide a particular in-depth investigation of significant factors of a phenomenon. A convenient sampling technique based on purposive sampling was utilised in this study. To invite participants to the study, a research invitation was posted at the reception desk of the identified health centre with specific inclusion criteria. Only those who met the inclusion criteria were selected. The inclusion criteria were participants who must have used alternative medicine to treat their illness, have resided within a rural community, were above the age of 18 years and were willing to participate in the study voluntarily. As soon as the participants who met the inclusion criteria agreed to participate in the study, the researcher invited the participants to participate in an online text-based interview via e-mail. Consent forms were obtained. This approach was most favourable as it avoided contact with the participants, thus minimising the spread of the COVID-19 virus. Saturation was achieved when the researchers received the tenth feedback from the participants. The researchers then began to transcribe, code and analyse the data. The participants included six male and four female patients from Gauteng province between October and November 2021 (see Table 1).

**TABLE 1:** Participants’ information.

Participants	Gender	Age
P1	Male	45
P2	Female	51
P3	Female	35
P4	Male	38
P5	Male	40
P6	Male	69
P7	Male	25
P8	Female	53
P9	Female	48
P10	Male	30

Data collection is a systematic process of gathering information to answer research questions and evaluate outcomes (Creswell 2014; Yin 2018). The researcher used the text-based open-ended interview to elicit information on participants’ experiences of the use of TCM. The data were analysed using a thematic approach (Creswell 2014). Both Maree (2020) and Yin (2018) specified thematic analysis as a process of identifying similar and dissimilar opinions with qualitative data. This will assist researchers in making sense of the data and identifying essential aspects of the research. Therefore, the six-step framework of thematic analysis proposed by Creswell (2014) was followed in this study. To ensure that the data were correct and without misinterpretation, post-transcription and member checking took place (Creswell 2014). The ethical consideration of this article includes obtaining informed consent and maintaining anonymity, confidentiality and privacy, as well as avoidance of betrayal and deception to meet the requirements of the ethical code of conduct. A second coder reviewed the raw data and themes to ensure the neutrality of the findings.

### Ethical considerations

Ethics clearance was obtained from a Research Ethics Committee at a public university in Gauteng province (reference number: REC-1233-2021). To ensure the anonymity of the 10 participants, the numerical codes P1–P10 were used.

## Results: Findings and discussion

In reviewing related research manuscripts to determine whether the use of TCM can improve the rural health in Africa, three themes emerged through the coding and data analysis, given as follows: (1) the attitude of TCM amongst rural African populations; (2) the advantages of using TCM in rural areas; and (3) the use of TCM therapeutic techniques.

### Theme 1: The attitude to traditional Chinese medicine in rural population

Rural health is crucial globally, and the population in rural areas is more open to alternative medicine (Tlou 2018; Wardle et al. 2011). This study revealed that the attitude towards alternative medicine amongst rural residents plays a vital role in its application and use as a choice. All participants in this study were open to TCM and were willing to utilise TCM as a form of treatment for their health. A participant stated that:

‘Traditional Chinese medicine is similar to African herbal medicine amongst the African people. The medicine that the doctor prescribed were all from plant matter; therefore, it is not different from my culture.’ (P1, Male, 45 years old)

Another response to TCM was:

‘The herbal medicine is no different from the herbs that our traditional healer gives us to drink. We grew up with herbal medicine, and therefore, our people know and understand the importance of herbal medicine, which you call alternative medicine.’ (P3, Female, 35 years old)

Similar sentiments were reported by many authors who have paid particular attention to the rural population’s attitude to alternative medicine. For example, both Shumer et al. (2016) and Wardle et al. (2011) asserted that rural populations are more open to alternative medicine in Japan and Australia. Another study conducted in rural China obtained similar findings that the rural residents prefer to use TCM for chronic diseases (Liu et al. 2008). P4 (Male, 38 years old), P5 (Male, 40 years old) and P7 (Male, 25 years old) agreed that:

‘They grew using herbal medicine throughout their childhood. They indicated that their parents would send them out into the garden to dig up ginger bulbs and African potatoes and collect different herbs for the sick patient at home. These were boiled or grounded and given to those who suffered severe coughs, colds and other illnesses.’

Studies have shown that millions of people in Africa are open to alternative medicine, as reported by Amzat and Razum (2018), Maroyi (2013) and Wassie et al. (2015). Therefore, there is a high probability that Africa’s rural residents’ attitudes will be favourable towards TCM.

In response to the question on their perception of the use of TCM, P9 (Female, 48 years old) and P10 (Male, 30 years old) stated that:

‘For any person who wants to use alternative medicine, they first must have some knowledge and understanding of the values and properties of the medication. The medical practitioner must explain the medication before the patient begins to use it. People should be made educationally aware of the properties of the alternative treatment.’

The knowledge of TCM is of paramount importance for the choices of patients when they are selecting medical assistance (Wardle et al. 2011). Li et al. (2015) argued that patients with high education levels are more likely to use TCM. This claim is in agreement with the study conducted by Shumer et al. (2016) in Japan. We believe that residents with better education may have a better understanding of TCM. Therefore, the introduction of TCM knowledge to rural residents becomes necessary. According to the HBM, people will improve their health only if they understand that they can be affected (perceived susceptibility) and the severity of the disease (perceived severity). They will also need to realise that their health may benefit from taking actions to prevent or treat conditions (perceived benefits). The researchers of this study are of the view that these aspects will be strengthened by introducing TCM knowledge to rural residents.

### Theme 2: The advantages of using traditional Chinese medicine in rural areas

Traditional Chinese medicine is widely used as a treatment for various diseases worldwide, according to Kim et al. (2018) and Lam et al. (2019). The findings of this study were supported by other researchers that TCM can treat a wide range of diseases with confirmed effectiveness and low cost in different counties. To this, a participant stated:

‘I was told by the Chinese medicine doctor that I could cook spring onions and fresh ginger to treat the common cold. I have tried it and it worked.’ (P2, Female, 51 years old)

Another participant added:

‘I used to use ginger mash for my arthritis. The pain was reduced significantly.’ (P6, Male, 69 years old)

Similar findings were reported by Schlabach et al. (2012) and Venzke et al. (2010) as mentioned in the literature.

Wang et al. (2015) claimed that the TCM can effectively improve patients’ conditions with metabolic syndrome from a study with 598 participants in China. Ren et al. (2014) carried out a randomised, double-blinded controlled trial with moxibustion on patients with chronic knee osteoarthritis. Patients in the treatment group experienced pain reduction, function improvement and stiffness decrease compared with the controlled group. Arslan et al. (2015) argued that cupping may be considered for all musculoskeletal pain conditions. This suggestion aligns with Coyle (2010), who agreed that pain is the most common reason people seek alternative medicine. As stated above, TCM has been widely used in different contexts worldwide for various diseases at affordable costs. The implementation of TCM in rural communities will improve the quality of their health.

According to Eaton et al. (2018), the rural population is less likely to use self-management interventions than the urban population. Similar sentiments were identified in our study. All participants reported that they would seek medical assistance only when they experienced ailments they could not tolerate. When answering their views on health, a participant said:

‘I have never monitored my blood pressure and blood sugar. I don’t see doctors for general health check-ups. I will only see them when I am badly sick. I don’t think that I need to do anything to my health when I am healthy.’ (P8, Female, 53 years old)

Another participant stated that:

‘As a pensioner, I could not afford medical services as they are just too expensive for me. I don’t know how to manage my health.’ (P6, Male, 69 years old)

As specified by Harris et al. (2011), one of the common reasons for delays in seeking medical assistance is a lack of awareness of the severity of diseases. Tarkang and Zotor (2015) supported that the HBM can be used for health promotion. Coyle (2010) conducted a study in a rural area in south-eastern Victoria, Australia; the findings revealed that 31% of residents were referred through word of mouth. In response to how participants knew of TCM, P1 (Male, 45 years old) indicated, ‘I heard of traditional Chinese medicine from a friend of mine who went for acupuncture treatment to a Chinese doctor in Johannesburg.’ According to P3 (Female, 35 years old) and P6 (Male, 69 years old), they heard of Chinese medicine from their families and friends’ children who were studying homoeopathy at the university. These findings correlate with the literature by Coyle (2010), where it appears that word of mouth is significant advocacy for the use of TCM.

In South Africa, TCM has been gaining recognition because it was formally introduced as part of a degree programme (Traditional & Natural Health Alliance 2018); the researcher believes that teaching TCM to rural residents is essential and affordable to help people engage in self-care and maintain their health. According to HBM, to promote healthy behaviours and decisions, individuals must believe that there are risks of being sick (perceived susceptibility) and understand the severity of the diseases (perceived severity) – therefore, the use of appropriate alternative treatment is crucial. These two aspects, without a doubt, can be achieved by introducing general health knowledge of TCM. After understanding basic TCM knowledge, the belief in efficacy (perceived benefits) will be achieved by understanding diseases and health knowledge.

### Theme 3: The use of traditional Chinese medicine therapeutic techniques

As revealed by Shen et al. (2010), applying correct Chinese food therapy in diet may reduce blood pressure in patients with hypertension. According to P2 (Female, 51 years old) and P8 (Female, 53 years old), they both indicated that they watched their diet and ate recommended foods. This has helped them to bring down their blood pressure. This claim is in agreement with Wang et al. (2017), after the implementation of ‘simple therapeutic techniques of TCM’ in the rural areas in China, the number of patients’ consultations was reduced by 34% after a two-year follow-up (*p* < 0.05). The content of the ‘simple therapeutic techniques of TCM’ focuses on the TCM health education and convenient treatment techniques that can be performed by residents themselves without comprehensive training, such as food therapy, moxibustion, cupping and Chinese therapeutic massage. It is a unique approach from a holistic perspective of TCM that applies interventions from multiple aspects to maintain health (Li et al. 2019). Findings from this study agreed with these authors as participants asserted that some simple techniques would benefit their health. For example, a participant stated:

‘I cook ginger slices and brown sugar together whenever I experience the period pain. I have been using this method for quite a few years. I am sure it works very well.’ (P9, Female, 48 years old)

Both Mellor (2014) and Tlou (2018) argued that the accessibility and affordability of alternative medicine, such as acupuncture, cupping and massage, are important reasons for patients’ choices. We believe that the rural residents in Africa will benefit significantly from this kind of holistic approach in both treating and preventing diseases, especially at no or low extra cost.

In a similar vein, Wassie et al. (2015) argued that the knowledge of TM can potentially assist in selecting plant materials and discovering their therapeutic effects. Wassie et al. (2015), in a study conducted in Ethiopia, further highlighted that 90.3% of the participants would like to acquire knowledge of herbal medicine. We believe that employing TCM theories to analyse African medicinal plants will broaden their actions and indications. After gaining basic knowledge of TCM, the rural residents in Africa will identify different functions of herbs to treat diseases or maintain health by themselves.

With a deep understanding of the advantages of TCM, rural residents may be encouraged to accept multiple health behaviours, including diet changes, the use of herbal medicine, moxibustion, cupping and massage. According to the HBM, rural communities also like to enjoy good health. They are eager to try alternative medicine, even visiting herbalists. Self-efficacy, according to the HBM, which refers to the ability to conduct action, will also be encouraged. Furthermore, the number of qualified TCM service providers is essential for long-term implementation; as argued by Liu et al. (2008), most residents in rural areas prefer to have TCM treatment performed by qualified TCM practitioners. We argue that training for different levels of TCM service providers is encouraged for the specific need of rural health in Africa. This can be supported by the successful experience of ‘barefoot doctors’ who were specifically trained for the market of rural health in China in the mid-twentieth century (Xu & Hu 2017).

## Recommendations

Rural health in Africa is critical because of multiple factors, for example, the lack of health knowledge and healthcare service providers, socio-economic conditions and poverty. To improve rural health in Africa, the following recommendations are proposed:

- introducing basic TCM knowledge to the rural population, including correct diet from a TCM perspective
- promoting TCM health education amongst the rural population, as well as the application of TCM theories in analysing the functions of African medicinal plants
- introducing basic and simple TCM treatment techniques to the rural residents, including herbal remedies, moxibustion, Chinese therapeutic massage and cupping
- promoting and improving basic TCM treatment techniques to healthcare service providers in rural areas, in the forms of formal and informal conferences and training
- educating more TCM service providers specifically for the needs of rural health, such as acupuncturists.

## Conclusion

Poverty, lack of health knowledge and TCM service providers in rural areas have made the call for improving rural health in Africa critical, considering the current poor health situation. There is a need to seek other safe, efficacious and cost-effective medical services in Africa, primarily in rural areas. Traditional Chinese medicine is widely used in treating a wide range of diseases worldwide, both in urban and rural areas. The use of TCM may improve rural health in African countries because of several advantages of TCM, such as the low cost and effectiveness. One of the limitations of this study is from the HBM, which does not consider behaviours that are performed for non-health-related reasons such as social acceptability. Further studies are recommended to develop a more comprehensive theoretical framework to analyse health behaviours. There is also a lack of research from the literature on the African population’s views of TCM; further studies on residents’ experiences and perceptions of TCM are recommended.
